# Sublethal dose of irradiation enhances invasion of malignant glioma cells through p53-MMP 2 pathway in U87MG mouse brain tumor model

**DOI:** 10.1186/s13014-015-0475-8

**Published:** 2015-08-06

**Authors:** Jian Pei, In-Ho Park, Hyang-Hwa Ryu, Song-Yuan Li, Chun-Hao Li, Sa-Hoe Lim, Min Wen, Woo-Youl Jang, Shin Jung

**Affiliations:** Department of Neurosurgery, Chonnam National University Hwasun Hospital and Mediacal School, 322 Seoyang-ro, Hwasun-eup, Hwasun-gun, Jeonnam, 519-763 Republic of Korea; Brain Tumor Research Laboratory, and Chonnam National University Research Institute of Medical Sciences, Chonnam National University Hwasun Hospital and Medical School, 322 Seoyang-ro, Hwasun-eup, Hwasun-gun, Jeonnam, 519-763 Republic of Korea

**Keywords:** Radiation, Glioblastoma, p53, MMP-2, TIMP-2, Sublethal dose

## Abstract

**Background:**

Glioblastoma is a highly lethal neoplasm that frequently recurs locally after radiotherapy, and most of these recurrences originate from near the irradiated target field. In the present study, we identified the effects of radiation on glioma invasion and p53, TIMP-2, and MMP-2 expression through *in vitro* and *in vivo* experiments.

**Methods:**

The U87MG (wt p53) and U251 (mt p53) human malignant glioma cell lines were prepared, and the U2OS (wt 53) and Saos2 (del p53) osteosarcoma cell lines were used as p53 positive and negative controls. The four cell lines and p53 knock-downed U87MG cells received radiation (2–6 Gy) and were analyzed for expression of p53 and TIMP-2 by Western blot, and MMP-2 activity was detected by zymography. In addition, the effects of irradiation on directional invasion of malignant glioma were evaluated by implanting nude mice with bioluminescent u87-Fluc *in vivo* followed by MMP-2, p53, and TIMP-2 immunohisto-chemistry and *in situ* zymography.

**Results:**

MMP-2 activity and p53 expression increased in proportional to the radiation dose in cell lines with wt p53, but not in the cell lines with del or mt p53. TIMP-2 expression did not increase in U87MG cells. MMP-2 activity decreased in p53 knock-downed U87MG cells but increased in the control group. Furthermore, radiation enhanced MMP-2 activity and increased tumor margin invasiveness *in vivo*. Tumor cells invaded by radiation overexpressed MMP-2 and p53 and revealed high gelatinolytic activity compared with those of non-radiated tumor cells.

**Conclusion:**

Radiation-induced upregulation of p53 modulated MMP-2 activity, and the imbalance between MMP-2 and TIMP-2 may have an important role in glioblastoma invasion by degrading the extracellular matrix. Bioluminescent “U87-Fluc”was useful for observing tumor formation without sacrifice after implanting tumor cells in the mouse brain. These findings suggest that the radiotherapy involved field for malignant glioma needs to be reconsidered, and that future trials should investigate concurrent pharmacologic therapies that inhibit invasion associated with radiotherapy.

**Electronic supplementary material:**

The online version of this article (doi:10.1186/s13014-015-0475-8) contains supplementary material, which is available to authorized users.

## Background

Glioblastoma (GBM) is the most malignant of primary adult brain tumors and is characterized by a highly localized invasive cell population, abundantly proliferative cells, neoangiogenesis, and necrosis. The current treatment standard involves a multimodal approach of neurosurgery, fractionated radiotherapy (RT), and chemotherapy [1]. However, despite continuous improvements in GBM treatment, median survival time does not exceed 15 months [2].

RT is the main treatment for brain tumors. The RT target volume consists of the tumor volume with a 2–3 cm margin of surrounding tissue, which is considered to be at risk for microscopic tumor invasion. Traditional target-volume RT has raised a question of whether RT could be helpful for killing tumor cells that have infiltrated the surrounding normal brain. In fact, whole brain RT has not decreased the risk of recurrence and has seriously increased the risk of neuropsychological deficits, e.g., dementia [3]. Furthermore up to 90 % of all gliomas relapse in close proximity to the resection cavity or the postoperative RT target volume [4, 5]. In other words, it may be important to understand the biological characteristics of invading cells into the surrounding brain tissue. Invasive tumor cells have a G0 phase, are resistant to RT and chemotherapy, and eventually regrow. G0/G1 phase cells migrate more rapidly and further than cancer cells in the S/G2/M phases. Cancer cells cease migrating when they enter the S/G2/M phases and restart migration after cell division when they re-enter the G0/G1 phase [6]. Radiation to surrounding brain tissue augments secretion of several mediators, including stromal cell-derived factor-1α, vascular endothelial growth factor (VEGF) and matrix metalloproteinases (MMPs), which play a vital role in tumor invasion and metastasis [7].

MMPs are a family of zinc-dependent proteolytic endopeptidases that break down the structural barriers to migration and invasion by dissolving and destroying the matrix proteins of surrounding normal brain tissue [8, 9]. In 2001, Wild-Bode et al. reported that sublethal doses of radiation promote glioma invasion [10]. Another study suggested that this may be mediated by MMPs, such as MMP-2, as irradiation increases MMP-2 levels [11]. In particular, MMP-2 and 9 are highly expressed in GBM compared with that in normal brain tissue [1]. Ionizing radiation enhances MMP-2 production in a variety of human cancers, and activation of wild-type p53 by radiation and the resulting increase in MMP-2 expression suggest that radiation may promote invasion-related gene expression [12–15].

p53, a 53-kDa nuclear protein, is one of the most extensively studied molecules in cancer research and molecular biology. p53 has many anticancer functions and plays critical roles in DNA repair, apoptosis, and inhibiting angiogenesis [16]. Wild-type p53 is required to activate apoptosis in response to DNA damage [17] and, thus, may be a critical determinant of the effectiveness of ionizing radiation and chemotherapeutic agents. According to a 1995 report, patients with cancer harboring a p53 mutation often have poorer prognoses than those with tumors that harbor wild-type p53 [18]. Thus, the genetic and functional status of the p53 gene is an important factor guiding therapeutic strategies in patients with cancer [19]. In 1997, after a computer search for other potential p53 target genes, Bian et al. determined that wild-type p53 binds the promoter of the gene encoding human type IV collagenase (also called gelatinase A or MMP-2), a naturally occurring enzyme subject to inhibition by TIMP-2, among other known p53 target genes [14, 20].

In this study, we investigated the effects of radiation on malignant glioma invasion and p53, TIMP-2, and MMP-2 expression through *in vitro* and *in vivo* experiments. As focal brain irradiation remains the standard of care for managing malignant glioma, understanding the effects of RT on invasion of malignant glioma may impact RT strategy. We hope that our findings will help understanding the changes occurring in surrounding normal brain tissues within the postoperative RT field and provide a basis for explaining local relapse during or a few months after RT.

## Materials and methods

### Cell culture conditions

The human malignant glioma cell lines U87MG (wt p53) and U251 (mt p53) and the human osteosarcoma cell lines U2OS (wt p53) and SAOS (del p53) were acquired from the American Type Culture Collection (Manassas, VA, USA). The U87-Fluc cell line, which is transfected with a lentiviral vector containing the firefly luciferase (Fluc) gene, was a gift from Professor Min (Hwasun Chonnam National University Hospital, Korea). The cell lines were routinely maintained in high-glucose DMEM (Gibco BRL, Gaithersburg, MD, USA) supplemented with 10 % fetal bovine serum (Gibco) and maintained at 37 °C containing 5 % CO_2_/95 % air.

### siRNA oligonucleotide transfection

A siRNA oligonucleotide was used for p53 knockdown. The synthesized p53 siRNA (5′-CACUACAACUACAUGUGUA-3’) and scrambled RNA (negative control) were purchased from Bioneer (Daejeon Korea). Approximately 2 × 10^5^ u87MG cells were seeded on a plate and transfected with the siRNA oligonucleotide using Lipofectamine™ RNAiMAX (Invitrogen, Carlsbad, CA, USA), according to the manufacturer’s instructions. p53 knockdown was confirmed by Western blotting.

### Radiation

The cell lines were grown in 60 mm culture dishes until 80–90 % confluent and then the media were replaced with serum free media to synchronize the cell cycle. The cells were exposed to radiation from a Gammacell 1000 unit (^137^Cs; Nordion, Kanata, ONT, Canada) at 2, 4, and 6 Gy. The irradiated cells were further cultured for 24 h and harvested.

### Preparation of total protein and conditioned medium

The cells were lysed with a protein extraction buffer [50 mM Tris (pH 8.0), 5 mM EDTA, 150 mM sodium chloride, 0.5 % deoxycholic acid, 0.1 % sodium dodecyl sulfate (SDS), 1 % NP-40, 1 mM phenylmethylsulfonyl fluoride, and 1 mg/ml protease inhibitor cocktail] to prepare total protein. The cells were grown in 60 mm culture dishes until subconfluent, and the media were replaced with serum-free medium to prepare the conditioned medium. After radiation exposure, the cells were incubated at 37 °C for 24 h. The conditioned media were clarified by centrifugation, and protein concentrations were determined using a protein assay kit (Bio-Rad, Hercules, CA, USA).

### Western blot

Whole cell lysates (20 μg) were separated by 8–10 % SDS-polyacrylamide gel electrophoresis and transferred to PVDF membranes (Pall Corp., Port Washington, NY, USA). The membranes were incubated for 2 h at room temperature with 5 % non-fat dry milk, probed overnight at 4 °C with actin, p53, TIMP-2, and MMP-2 antibodies (Santa Cruz Biotechnology, Santa Cruz, CA, USA) and incubated with a horseradish peroxidase-labeled goat anti-rabbit IgG (Jackson Immunoresearch Laboratory, West Grove, PA, USA). Bound secondary antibody was detected by enhanced chemiluminescence (Amersham Biosciences, Bucks, UK), and protein levels were determined by autoradiography using a LAS-4000 instrument (Fuji, Tokyo, Japan).

### Gelatin zymography

Twenty μg of proteins in conditioned media were mixed with sample buffer (50 mM Tris–HCl, 2 % SDS, 0.1 % bromophenol blue, and 10 % glycerol) before electrophoresis. Aliquots were electrophoresed on 8 % SDS-polyacrylamide gels containing 1 mg/ml type A gelatin (Sigma-Aldrich, St. Louis, MO, USA). Each gel was washed three times for 30 min in 2.5 % Triton X-100 and then incubated for 20 h at 37 °C in incubation buffer [50 mM Tris–HCl (pH 7.5), 10 mM CaCl_2_, and 200 mM NaCl]. The gels were stained with Coomassie Brilliant Blue R-250 (0.2 % Coomassie Brilliant Blue R-250, 20 % methanol, 10 % acetic acid in water) and then destained in 20 % methanol and 10 % acetic acid in water.

### In vivo studies

Five- to six-week old male BALB/c athymic nu−/nu − mice (body weight, 20–30 g) were purchased from the Orient Co. (Seongnam, Korea). They were housed in groups of three or four under standard conditions at a temperature of 22 °C and a 12-h light/12-h dark cycle. The mice had free access to standard food pellets and tap water. The mice were anesthetized with isoflurane (2 %), and a mixture of ketamine (200 mg/kg) and xylazine (10 mg/kg) for irradiation. Radiation treatment was carried out 14–21 days after tumor implantation. Tumor growth was monitored twice weekly by optical bioluminescence imaging, and signals were observed in all animals 7 days after tumor implantation. All animal care, experiments, and euthanasia were performed in accordance with protocols approved by the Chonnam National University Animal Research Committee (Gwangju, Korea).

### Bioluminescence imaging

Anesthetized mice were placed in the light-tight chamber of the IVIS 100 imaging system (Caliper, Newton, MA, USA), equipped with a cooled charge-coupled device camera to obtain the tumor bioluminescence images. Photons emitted from the luciferase-expressing tumor were collected and integrated over 1-min periods. Pseudocolor images indicating photon counts were overlaid on photographs of the mice using Living Image software v. 2.25 (Caliper). A region of interest was selected manually based on signal intensity. The area of the region of interest was kept constant, and intensity was recorded as maximum radiance within each region of interest.

### Radiotherapy

RT was commenced 2 weeks after tumor implantation. A 6-MV X-ray was used via a linear accelerator (CLINAC 21EX; Varian, Palo Alto, CA, USA). The source to skin distance was 97.5 cm with a field size of 5 × 5 cm^2^ and a dose rate of 3 Gy/min. Water equivalent boluses of 1 cm thickness were placed under and above the tumor-bearing mouse brain to establish dose homogeneity. Additional file 1: Figure S1 illustrates the process to assess the radiation effect on brain tumors *in vivo*. After implanting the U87-Fluc cells in the brain, tumor bioluminescence of mouse brain cells bearing U87-Fluc was observed continuously from day 10 to 28 using the IVIS 100 imaging system. Radiation exposure was either a single 6 Gy dose or 2 Gy fractions five times for 7 days on day 14 after U87-Fluc implantation. The mice brain tissue was stained with hematoxylin and eosin (H&E) or immunohistochemical staining after killing the mice on day 30.

### Histopathology

All mice were anesthetized and perfused transcardially with 4 % ZBF-containing 36.7 mM ZnCl_2_, 27.3 mM ZnAc_2_ · 2H_2_O, and 0.63 mM CaAc_2_ in 0.1 M Tris, pH 7.4. The brain tumor was removed, fixed in the same solution for 36–38 h at room temperature and then dehydrated for paraffin embedding. The tumors were blocked in cross-section and processed for paraffin embedding.

Four μm-thick consecutive sections were cut from the recipient blocks and placed on poly-L-lysine-coated slides for immunohistochemistry. Representative sections were stained with H&E. Heat-induced epitope retrieval was carried out for 10 min at 120 °C in a pressure cooker in 10 mM citrate buffer, pH 6.0 (Tris–EDTA buffer, pH 9.0 was used to detect MMP-2). Endogenous peroxidase activity was blocked by incubating the samples in PBS containing 3 % H_2_O_2_, and the non-specific binding sites were blocked with 3 % bovine albumin (Sigma-Aldrich) in PBS for 20 min at room temperature. The following primary monoclonal antibodies were added: MMP-2 (1:8000; Lab Vision Corp. Fremont, CA, USA), glial fibrillary acidic protein (GFAP) (1:4000, Lab Vision Corp), and p53 (1:8000; Santa Cruz Biotechnology). Then, a biotin-labeled secondary antibody (Dako, Carpinteria, CA, USA) was added, and the samples were incubated at room temperature for 1 h. A streptavidin-horseradish peroxidase (Dako) detection system was applied to the capillary channels, followed by a 20 min incubation at room temperature. The tissue sections were ready for the chromogen reaction with diaminobenzidine. Counterstaining was performed with Harris hematoxylin. Control experiments were performed in all specimens at the time of immunostaining using the secondary antibody alone.

### In situ zymography

*In situ* zymography was performed to localize gelatinolytic activity, essentially as described by Sbai et al. [21] and Miller et al. [22]. Eight-mm-thick sections were cut from the same tissue used for immunohistochemistry. The fixed, paraffin-embedded tissue sections were heated to 59 °C overnight, deparaffinized in xylene, and rehydrated in a graded alcohol series. The substrate was prepared by dissolving 1 mg DQ gelatin in 1 ml Milli-Q water, followed by a 1:50 dilution in a reaction buffer containing 50 mM Tris–HCl, 150 mM NaCl, 5 mM CaCl_2_, and 0.2 mM sodium azide (pH 7.6). Of this mixture, 250 μl was placed on top of the tissue sections, covered with Parafilm, and incubated in a dark humidity chamber at 37 °C. The Parafilm was removed gently after 2 h, and the sections were rinsed with Milli-Q water and fixed in 4 % neutral-buffered formalin for 10 min in the dark. Sample autofluorescence was quenched in a 100 mg/ml NaBH_4_ solution for 30 min. The sections were rinsed in a PBS bath (2 × 5 min) and mounted with glycerol containing DAPI to counterstain the nuclei. Control slides were pre-incubated with 20 mM EDTA for 1 h to verify the contribution of MMPs. A 20 mM EDTA solution was also added to the substrate. Tissue autofluorescence level was evaluated by incubating control sections at 220 °C for 2 h immediately after the substrate had been added. Fluorescence was evaluated using an Olympus confocal laser microscope with Olympus Application Suite Advanced Fluorescence software (Tokyo, Japan).

### Statistical analyses

Each experiment was repeated at least three times to confirm reproducibility. Comparisons were made using one-way analysis of variance. Differences between means were evaluated using the Kruskal–Wallis test, and p-values < 0.05 were considered significant.

## Results

### Effect of radiation on MMP-2 activity according to p53 status

**Fig. 1:**
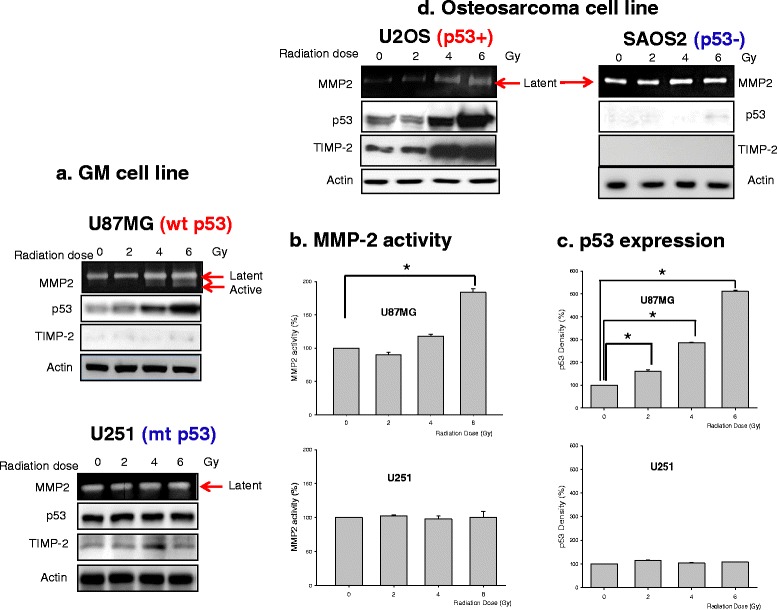
Radiation effect on p53, TIMP2 expression and MMP-2 activity in 2 cell lines with wt p53 and 2 cell lines without p53. Each cell lines were exposed 0, 2, 4, 6 Gy, respectively. MMP-2 activity was detected using zymography (*one lane*) and expression of TIMP-2 and p53 was detected using western blot (*2*–*4 lane*) **a** Glioblastoma cell lines; **b** MMP-2 activity of U87MG and U251; **c** p53 density of U87MG and U251; **d** Osteosarcoma cell lines

The human malignant glioma cell lines U87MG (wt p53) and U251 (mt p53) and the human osteosarcoma cell lines U2OS (wt p53) and SAOS (del p53) were selected to measure the effect of radiation on p53 expression and MMP-2 activity. As shown Fig. 1, MMP-2 activity and p53 expression increased in U87MG and U2OS cells wt p53 in proportion to the radiation dose, but these were unchanged in U251 and SAO2 cells. TIMP-2 is an endogenous MMP-2 inhibitor, and the balance between TIMP-2 and MMP-2 is a critical determinant of malignant glioma invasion. We performed a Western blot for TIMP-2 in the four cell lines exposed to radiation. TIMP-2 expression did not change after radiation exposure in three of the cell lines, except U2OS. The sublethal radiation dose increased p53 expression and MMP-2 activity in U87MG cells without a corresponding increase in TIMP-2 (*p* < 0.05, Fig. 1a, b, c). In contrast, TIMP-2 expression and MMP-2 activity in U2OS cells with wt p53 increased simultaneously following radiation. These results suggest that TIMP-2 expression was likely to be different depending on the cell type, and that the imbalance between TIMP-2 and MMP-2 expression may enhanced tumor invasion. In addition, MMP-2 activity caused by radiation may be associated with p53 status.

### Radiation-induced p53 upregulation is responsible for promoting MMP2 activity

**Fig. 2:**
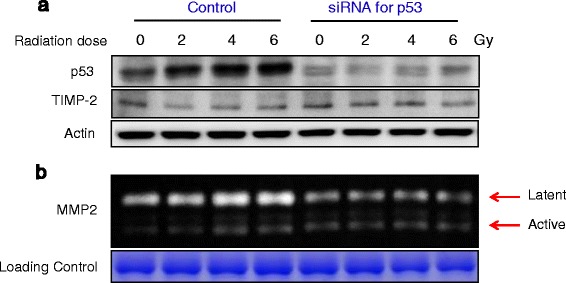
Radiation effect on p53, TIMP2 expression and MMP-2 activity in p53 knockdown U87MG. ‘Control’ Scramble RNA transfected U87MG, ‘siRNA for p53’ is p53 knock-downed U87MG using siRNA technology. **a** Western blot; **b** Zymography for MMP-2 activity

MMP-2 activity and p53 expression simultaneously increased after radiation exposure, and the MMP-2 gene promoter region has a putative p53 binding site. Thus, we identified whether p53 actually enhanced radiation-induced MMP-2 activity. U87MG was transfected p53 siRNA oligonucleotide and was performed Western blot for p53 and TIMP-2 (Fig. 2a), and Zymography for determining of MMP-2 activity (Fig. 2b). p53 expression and MMP-2 activity increased in proportion to the radiation dose in control transfected scramble RNA to U87MG cells, but remained unchanged in p53 knockdown U87MG cells (Fig. 2a, b). TIMP-2 expression was not changed by radiation, and no difference in TIMP-2 expression was detected between the control and p53 knockdown U87MG cells.

### Establishment of a bioluminescent tumor cell line

**Fig. 3:**
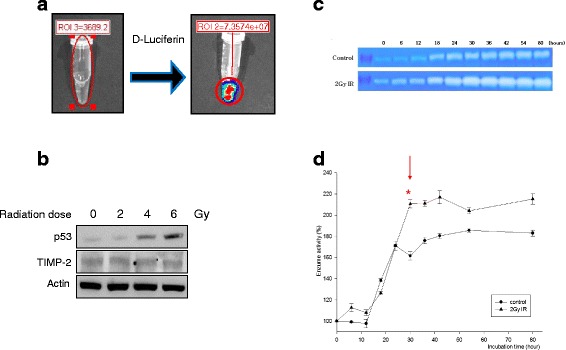
Radiation effect on p53, MMP-2 and TIMP-2 in U87 MG-Fluc cells. Establishment bioluminescent tumor cells “U87-Fluc” which is transfected with firefly luciferase (fluc) in u87MG. **a** Photons emitted from U87-Fluc cell line when D-luciferin was added; **b** Western blot; **c** Zymography for detection MMP-2 activity in dependent on time; **d** Density of MMP-2 activity. It had been showed statistical significance between control and 2 Gy IR from 30 hr (*p* < 0.05)

We established the bioluminescent tumor cell line “U87-Fluc” for *in vivo* experiments, in which u87MG was transfected with firefly luciferase (Fluc). This is a useful tool to observe tumor formation without sacrifice after implanting tumor cells in the mouse brain. The U87-Fluc cell line emitted photons when D-luciferin was added (Fig. 3a), and U87-Fluc cells increased p53 expression and MMP-2 activity in proportion to the radiation dose but did not affect TIMP-2 expression (Fig. 3b, c). MMP-2 activity increased depending on the incubation time after radiation exposure and showed a great difference between control and radiated cells after 24 hr (*p* < 0.05, Fig. 3d).

### Tumor growth rate analyses using optical bioluminescence imaging

**Fig. 4:**
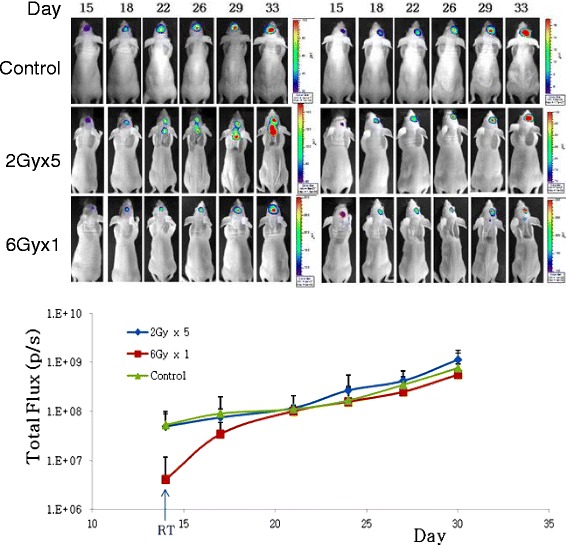
Tumor growth rate analyses using Optical Bioluminescence Imaging. U87-Fluc bearing nude mice brain were continuously observed through tumor bioluminescence by the IVIS 100 imaging system. For pilot study we designated control group (non-radiated group) and two groups (radiated group). Among radiated group, one group was exposed to fractionated dose radiation (2Gy x 5) and another group was exposed to single dose radiation (6 Gy)

U87-Fluc-bearing nude mice brains were observed continuously by tumor bioluminescence using the IVIS 100 imaging system to determine the effects of radiation on tumor growth. Additional file 1: Figure S1 illustrates the process used to assess the radiation effects on brain tumors *in vivo*. The mouse brains were exposed to radiation after tumor formation had been observed by tumor bioluminescence. As shown in Fig. 4, notable retardation of tumor growth was observed in the single 6 Gy exposed group, compared with that in the control groups. However, the brain tumors continued to grow, and the final growth rate was similar to that of the control group from about day 20. No difference was found in the radiation-fractionated group, compared to the control group, indicating that the single dose (6 Gy) treatment or low dose fractionated irradiation was insufficient for complete control of tumor growth.

### Irradiation promotes glioma cell dissemination in vivo

**Fig. 5:**
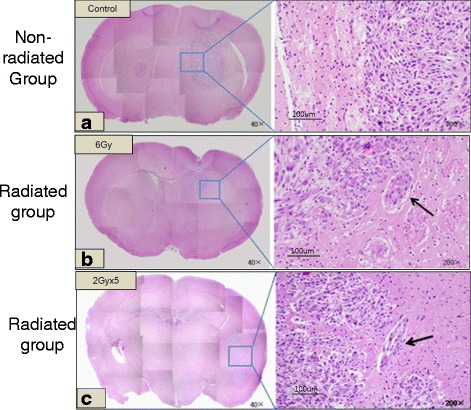
Hematoxyin and Eosin staining of brain tissues. Exposure to radiation was more invasive and exhibited multiple tumor cell satellites, while non-radiated tumor showed less invasiveness and clear margin. **a** Non-radiated group, **b** Radiated group (Single dose), **c** Radiated group (fractionation)

The observed invasive effect of sublethal irradiation in U87-Fluc cells *in vivo* may have clinical relevance. As shown in Fig. 5 tumors in the non-radiated group had clear borders, whereas in the radiated group showed more irregular margin, the diffuse infiltration of tumor cells into surrounding normal brain and exhibited multiple tumor satellites.

**Fig. 6:**
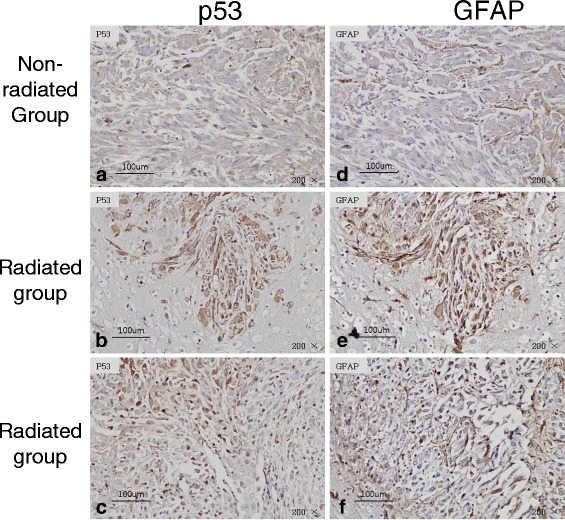
Immunohistochemistry for p53 and GFAP in brain tissues. GFAP (**d**,**e**,**f**) as a glial cell marker was stained to confirm whether p53 (**a**,**b**,**c**) positive cells were glioma cell or not

The *in vitro* results showed an association between p53 and MMP-2. We wanted to confirm whether tumor cells invading after radiation exposure was related with p53 expression and MMP-2 activity. Thus, we verified that the invasive cells surrounding normal brain were malignant glioma cells using GFAP staining as a glial cell marker, and performed immunohistochemistry for p53 and MMP-2. p53 was highly expressed in irradiated tumors and was particularly distributed in invasive tumor cells around the margin area (Fig. 6), even though no difference was observed between the single and the fractionated dose groups. GFAP and p53-positive cells were distributed similarly.

**Fig. 7:**
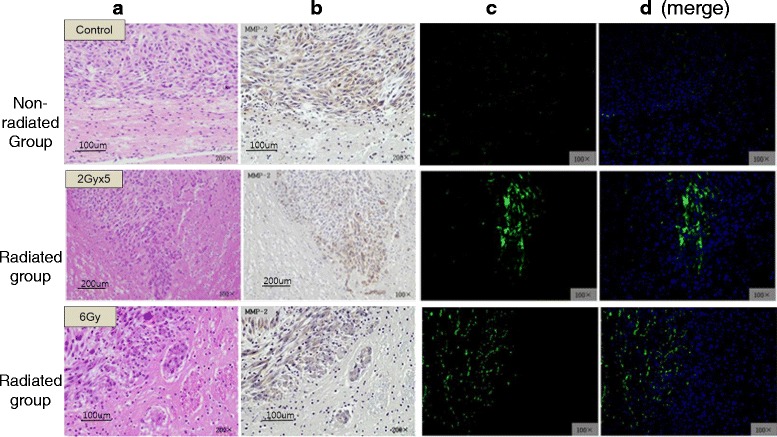
Immunohistochemistry for MMP-2 expression and in situ zymography for gelatinolytic activity in brain tissues with either radiation exposure or not. Autofluorescence of mice in the in situ zymography experiment was minimized by NaBH4. **a** Hematoxylin-Eosin stain; **b** Immunohistochemistry for MMP-2; **c**, **d** In situ zymography, green: gelatinolytic activity, blue: nucleus

*In situ* zymography was performed in radiation-exposed and unexposed nude mouse brains. This is a unique technique that enables localization of gelatinolytic activities in histological sections. Notably, autofluorescence during *in situ* zymography was minimized by NaBH4. Green fluorescence as a signal for gelatinolytic activity was found around the tumor margin area in the radiated group but not in non-radiated group (Fig. 7c, d). However, MMP-2 expression showed no difference between the two groups (Fig. 7b).

## Discussion

Primary GBM represents about 90 % of GBMs, and the wild-type p53 gene is present in about 70 % of primary GBMs. However, p53 occurs less frequently in secondary GBMs (35 %) [23]. p53 mutations frequently occur in low grade gliomas (WHO grade II astrocytoma) [24] and, thus, are a frequent event in the pathological progression of secondary GBM (WHO Grade IV) [25]. Secondary GBM arises from a preexisting grade II or III astrocytoma, whereas a primary GBM forms *de novo*. p53 mutations were detected in only 40 % of the samples, predominantly in anaplastic astrocytomas, indicating that >50 % of patients have wild type p53 [21].

In this study, we identified RT effect on migration and invasion of malignant glioma and studied the association between p53 and MMP-2 through in vitro and in vivo study. p53 is well known as a radio-sensitizer and radiation induces p53 expression. And it has been hypothesized that RT may promote tumor metastasis in a subset of human cancers harboring the wild-type p53 gene [3]. Increased p53 expression affects the metastatic potential of certain types of cancers [15, 22]. Radiation-induced p53 upregulation is responsible for promoting the VEGF-MMP2 pathway involved in enhancing the invasiveness of both irradiated and bystander hepatoma cells [26].

MMPs are a family of extracellular matrix (ECM)-degrading enzymes associated with numerous physiological and pathological events, such as malignant tumor cell invasion processes [8, 9]. And MMP activity is regulated at multiple levels, such as at the level of gene transcription and the synthesis of pro-MMPs. Ionizing radiation is known as a representative of DNA-damaging agents. The DNA cleavage by irradiation leads to the accumulation of p53 and its translocation into the nucleus [27, 28].

Then, the activated p53 binds to DNA in a sequence-specific manner and modulates a set of genes [29]. Other study has demonstrated that the promoter region of the MMP-2 gene has a putative p53 binding site and that wild-type p53 transcriptionally upregulates the expression of MMP-2 mRNA [14, 22]. Considering the above references, we examined the radiation effect on MMP-2 activity and association between p53 status and MMP-2 activity. In our study, MMP-2 activity was actually increased depending on the radiation dose in cell lines with wt p53 gene; however, no change of MMP-2 activity was shown in cell lines with mt p53 gene. So we expected that p53 increased by radiation could upregulate MMP-2 activities, then confirmed their association using p53 knock-downed u87MG cells by siRNA technology. MMP-2 activity was decreased regardless of the radiation dose in p53 knockdown, while increased depending on the radiation dose in the control. Even though further studies are required to identify direct association between p53 and MMP-2 activity, p53, at least, can modulate the change of MMP-2 activity by radiation on the basis of the present study.

Many studies have reported the effects of radiation on tumor cell migration, invasion, and surrounding healthy tissue. Radiation enhances cell invasion and induces MMP-2 and −9 activities in various tumors and in lung epithelial cells [30–32]. Surrounding the brain tissue exposed to radiation secretes several mediators, such as VEGF, MMPs, cyclooxygenase-2, and transforming growth factor-beta, all of which play vital roles in tumor invasion and metastasis [26, 33, 34]. An imbalance between MMP-2 and TIMP-2 has an important role in glioma invasion by degradation of the ECM [34, 35]. TIMP-2 is an endogenous MMP-2 inhibitor and critical for maintenance of tissue homeostasis by suppressing proliferation of quiescent cells in response to angiogenic factors, and by inhibiting protease activities in tissues undergoing ECM remodeling. Our results showed that the expression of TIMP-2 was not changed, while MMP-2 activity increased depending on the radiation dose in malignant glioma cell lines. We also obtained the same results through *in vivo* experiments. The radiation-exposed U87-Fluc-bearing mouse brain was significantly more invasive, while MMP-2 and p53 expression, as well as gelatinolytic activity, increased, particularly in margin areas. However, TIMP-2 expression increased along with MMP-2 activity in U2OS cells. Those results suggest that TIMP-2 expression was likely to be different depending on the organ specific cell type, and that the imbalance between TIMP-2 and MMP-2 in malignant glioma with wt p53 may be important to understanding radiation-enhanced glioma invasion. Finally, we confirmed that radiation could induce glioma invasion through in vivo brain tumor model using U87-Fluc cell with bioluminescent. The U87-Fluc-bearing mouse brain was observed continuously using tumor bioluminescence with the IVIS 100 imaging system. This is a useful tool to observe tumor formation without sacrificing animals after implanting tumor cells. As shown through the results, radiation significantly enhanced tumor cell invasion in the U87-Fluc-bearing mouse brain tumor model. We suggest that invasive brain tumor model caused by RT could be applied to in vivo studies for verification of the effect of anti-invasive therapeutic target genes or drugs.

The clinical implications of this study are as follows:

- Alterations of radiotherapy for human malignant glioma might be considered to determine whether the patient is harboring wild-type p53 or mutant type.
- We propose using an MMP inhibitor adjunctively to suppress the radiation-induced increase in MMP-2 expression and to prevent unexpected or unwanted tumor invasion due to RT.

## Conclusions

Radiation enhanced glioma invasion in the mouse brain tumor model. Wild type p53 induced by raidation modulated MMP-2 activity, and the imbalance between MMP-2 and TIMP-2 may have an important role in glioma invasion by degradation of the ECM. For the study using mouse brain tumor model, bioluminescent U87-Fluc was useful to observe tumor formation without sacrificing animals after implanting tumor cells. Taken together, these findings suggest that involved-field RT for malignant gliomas may need to be reconsidered. Future trials should consider pharmacological therapies that inhibit invasion concurrently with RT.

## Additional file

Additional file 1: Figure S1. The illustration of the experimental process to assess radiation effect on brain tumor in vivo. After implantation U87-Fluc cells into brain, tumor bioluminescence of mouse brain bearing U87-Fluc was continuously observed from day 10 to 28 using the IVIS 100 imaging system. Radiation was exposed to either Single dose 6Gy or 2Gy fractions 5 times for 7 days on 14 day after U87-Fluc implantation. Mice brain were performed with Hematoxylin and eosin or immunohistochemical staining after execution on 30 day. (PPTX 381 kb)

## Abbreviation

GBM: Glioblastoma
RT: RadioTherapy
wt: Wild type
mt: Mutant type
del: deletion
MMP: Matrix metalloproteinase
TIMP: Tissue inhibitor of Matrix metalloproteinase
EDTA: Ethylenediaminetetraacetic acid
