# Supplementary figures and images for: Sublethal dose of irradiation enhances invasion of malignant glioma cells through p53-MMP 2 pathway in U87MG mouse brain tumor model
Source: Radiat Oncol. 2015 Aug 6;10:164. doi: 10.1186/s13014-015-0475-8 (PMC4554349; doi:10.1186/s13014-015-0475-8)

## Slide 1
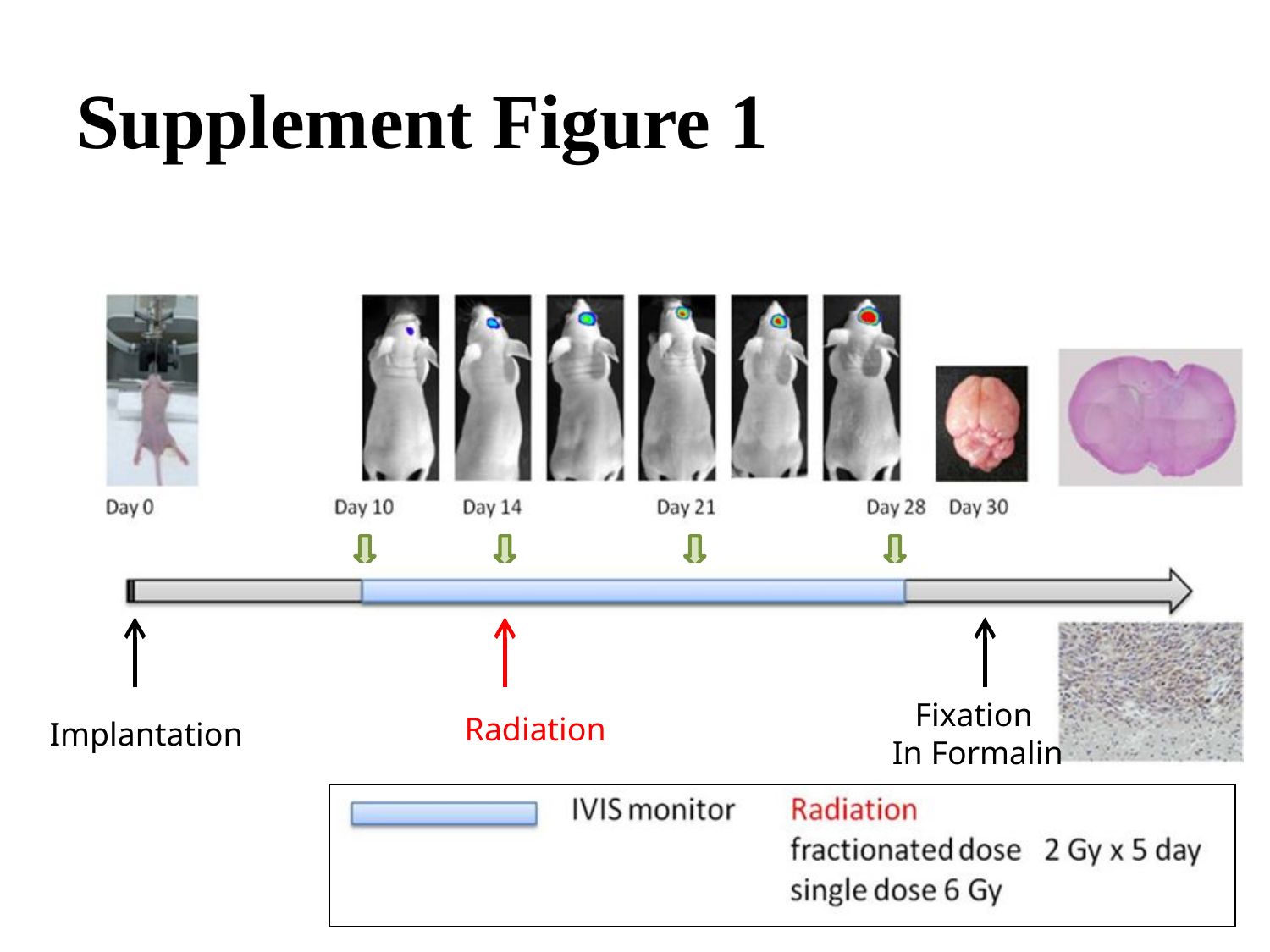

# Supplement Figure 1
Fixation
In Formalin
Radiation
Implantation

Supplement: Additional file 1: Figure S1. — The illustration of the experimental process to assess radiation effect on brain tumor in vivo. After implantation U87-Fluc cells into brain, tumor bioluminescence of mouse brain bearing U87-Fluc was continuously observed from day 10 to 28 using the IVIS 100 imaging system. Radiation was exposed to either Single dose 6Gy or 2Gy fractions 5 times for 7 days on 14 day after U87-Fluc implantation. Mice brain were performed with Hematoxylin and eosin or immunohistochemical staining after execution on 30 day. (PPTX 381 kb) [file 13014_2015_475_MOESM1_ESM.pptx]
